# The Effect of Iodinated Contrast Media Sensitivity on the Prognosis of Patients with STEMI

**DOI:** 10.3390/medicina60060973

**Published:** 2024-06-12

**Authors:** Alon L. Roguin, Edo Y. Birati, Ofer M. Kobo

**Affiliations:** 1 Azrieli Faculty of Medicine, Bar-Ilan University, Safed 5290002, Israel; alon.roguin@gmail.com (A.L.R.); ebirati@tzmc.gov.il (E.Y.B.); 2 The Kittner-Davidai Division of Cardiovascular Medicine and Surgery, Tzafon Medical Center, Ramat Gan 5290002, Israel; 3 Division of Cardiovascular Medicine, Hillel Yaffe Medical Center, Hadera 38100, Israel; 4 The Ruth and Bruce Rappaport Faculty of Medicine, Technion, Haifa 3109601, Israel

**Keywords:** allergy, contrast agents, mortality, myocardial infraction, national inpatient sample, percutaneous coronary intervention, prognosis, safety of treatment

## Abstract

*Background and Objectives*: Iodinated Contrast Media (ICM) is used daily in many imaging departments worldwide. The main risk associated with ICM is hypersensitivity. When a severe hypersensitivity reaction is not properly managed and treated swiftly, it may be fatal. Currently, there is no data to demonstrate how ICM sensitivity affects the prognosis of cardiac patients, especially those diagnosed with ST elevation myocardial infarction (STEMI), in whom urgent coronary angiography is indicated. This study aimed to identify and characterize this relationship. *Materials and Methods*: We included patients hospitalized with STEMI between 2016 and 2019 from the National Inpatient Sample. The population was compared based on ICM sensitivity status, sensitive vs. non-sensitive. The primary endpoint was in-hospital mortality, with additional endpoints: length of stay and in-hospital complications. *Results*: The study included 664,620 STEMI patients, of whom 4905 (0.7%) were diagnosed with ICM sensitivity. ICM-sensitive patients were older, more often white, females, and had more comorbidities and cardiovascular risk factors. Both groups show similarities in management but are slightly less probable to undergo PCI or CABG. Multivariable logistic regression models found that the ICM-sensitive population had similar odds of in-hospital mortality (OR: 1.02, 95% CI: 0.89–1.16) and MACCE (OR: 1.05, 95% CI: 0.95–1.16), and less major bleeding (OR: 0.73, 95% CI: 0.60–0.87). *Conclusions*: Our study found that ICM sensitivity status was not a significant factor for worse prognosis in patients hospitalized with STEMI.

## 1. Introduction

Patients diagnosed with ST-segment elevation myocardial infarction (STEMI) require urgent care to minimize mortality and irreversible cardiac damage. Urgent coronary catheterization and percutaneous coronary intervention (PCI) have shown significant improvement in mortality of STEMI patients [1]. Injection of iodinated contrast media (ICM) is essential during cardiovascular interventions providing real-time visualization of the coronary arteries and enabling angioplasty and stent placement. Currently, data regarding the effect of ICM sensitivity on the prognosis of patients with STEMI are limited.

ICM use poses risks, primarily associated with allergic responses, often presenting as hypersensitivity reactions (HSR). The American College of Radiology (ACR) defines and classifies HSR severity as follows [2]:
GradeAllergic-likePhysiologicMild: self-limiting without evidence of progressionlimited urticaria, cutaneous edema, limited “scratchy” throat, nasal congestion, conjunctivitisLimited nausea, transient flushing, headache, mild hypertension, spontaneous vasovagal reactionModerate: pronounced symptoms that usually require medical attentionDiffuse urticaria, diffuse erythema, stable vital signs, throat tightness w/o dyspnea, bronchospasm, mild to no hypoxiaProtracted nausea, hypertensive urgency, isolated chest pain, vasovagal reaction requiring and responding to treatmentSevere: Often life-threatening and can result in death or permanent morbidityDiffuse edema with dyspnea, Diffuse erythema with hypotension, laryngeal edema with stridor and/or hypoxia, bronchospasm, anaphylactic shockVasovagal reaction resistant to treatment, arrhythmia, convulsions, seizures, hypertensive emergency

Severe HSR has a reported incidence of 0.004% to 0.04% [3]. Premedication with antihistamines and corticosteroids is recommended to prevent such adverse reactions, and a 2022 meta-analysis found a significant reduction in adverse events with premedication [4,5].

HSR can be divided into two principal groups: immediate reactions (up to 1 h after ICM administration) and delayed reactions (1 h to 7 days after receiving ICM). Regarding the pathophysiology of immediate reactions, recent evidence has demonstrated the release of histamine and tryptase, suggesting the involvement of IgE antibodies in the immediate reactions [6]. Delayed reactions were linked to T-cell mediated pathogenesis through HLA molecules; these lymphocytes were found in the skin lesions of drug-induced maculopapular exanthema and other hypersensitive-induced skin lesions [7,8].

As mentioned above in the ACR classification, a severe HSR can have fatal manifestations in the cardiopulmonary system, with cardiopulmonary arrest being a nonspecific end-stage outcome, resulting from a combination of allergic-like and physiological reactions [2].

The management of known ICM-sensitive patients should include a comprehensive evaluation of the severity of the past reaction, including onset, duration, clinical manifestation, and specific agent used [2]. Premedication protocol should be followed if possible. Unfortunately, no significant work has been performed so far in the setting of managing an ICM-sensitive patient with acute STEMI.

Allergic angina (also known as Kounis syndrome) refers to an allergy-related acute cardiac syndrome, including stent thrombosis caused by anaphylactoid processes [9]. The phenomenon was mostly documented in individual case reports and case series [10,11]. A small human [12] and animal [13] investigation showed coronary spasm following intracoronary histamine injection, and an autopsy study found adventitial mast cells in a patient with variant angina [14]. However, it is still uncertain whether anaphylactoid mediators play a significant role in coronary spasms.

The impact of ICM sensitivity on the prognosis of patients with STEMI remains unexplored. This is a unique population as the interventional procedure is urgent and there is no time to wait with the antihistamines and corticosteroids premedication protocol. We hypothesized that STEMI patients with ICM sensitivity undergoing diagnostic or interventional procedures may experience worse clinical outcomes and higher mortality compared to non-sensitive patients. Understanding and anticipating these differences is crucial for tailored management, given the global prevalence of ICM-dependent procedures and the potential for severe [6], life-threatening reactions.

## 2. Materials and Methods

### 2.1. Data Source

The National Inpatient Sample (NIS) is the largest all-payer inpatient healthcare database in the US. It was developed by the Healthcare Cost and Utilization Project (HCUP) and is sponsored by the Agency for Healthcare Research and Quality (AHRQ). The NIS dataset includes information on 7 to 8 million hospital discharges annually since 2004. Starting in 2012, the NIS sample shows discharges from all hospitals participating in HCUP, representing a 20% stratified sample of all discharges from US community hospitals.

### 2.2. Study Design and Population

We analyzed all adult (≥18 years) patients hospitalized for STEMI during 2016–2019. ICD-10 CM codes were used to identify procedural information during hospitalization including invasive coronary angiography, PCI, coronary artery bypass graft (CABG) surgery, thrombolysis, use of mechanical ventilation, and circulatory support. Patient and procedural characteristics were extracted using ICD-10 codes provided in Supplementary Table S1. Clinical and demographic information were recorded for each hospital discharge including age, gender, race, admission day (weekday or weekend), expected primary payer, and median household income according to ZIP code. ICM-sensitive patient group was separated from the non-ICM-sensitive patient group using the ICD-10 code for Contrast agent allergy. Missing records for age, gender, elective and weekend admission, and mortality status were excluded from the analysis. Patients with type 2 MI or elective admissions were also excluded from the analysis. Each discharge record had information on up to 30 diagnoses.

### 2.3. Outcomes

The primary outcome was in-hospital mortality. Secondary outcomes examined included major adverse cardiac and cerebrovascular event (MACCE), acute ischemic cerebrovascular accident (CVA), and major bleeding, as well as length of stay. MACCE is defined as a composite of all-cause mortality, acute ischemic CVA or transient ischemic attack, and cardiac complications. Major bleeding events were defined as a composite of gastrointestinal, retroperitoneal, intracranial, and intracerebral hemorrhage, periprocedural hemorrhage, unspecified hemorrhage, or needing a blood transfusion, according to ICD-10 CM code as specified in Supplementary Table S1. Length of hospital stay was measured in days.

### 2.4. Statistical Analysis

Continuous variables were presented as a median and interquartile range, due to skewed data, and categorical data were presented as frequencies and percentages. Categorical variables were compared using the Pearson Chi-square test, while continuous variables were compared using the student’s *t*-test or the Kruskal–Wallis test, as appropriate. Sampling weights were used to calculate the estimated total discharges as specified by AHRQ. Univariable and multivariable logistic regression models were performed to examine the association between in-hospital outcomes and Contrast agent allergy, all expressed as odds ratios (OR) with corresponding 95% confidence intervals (CI). The models were adjusted for baseline differences between the groups: age, gender, race, admission day (weekday or weekend), expected primary payer, median household income according to ZIP code and medical record characteristics. Statistical analyses were performed on IBM SPSS version 26. Statistical significance was set at the 2-tailed 0.05 level, without multiplicity adjustment. All statistical analyses were performed on “IBM SPSS Statistics Version number 26” software.

### 2.5. Ethical Issues

The NIS database provides entirely anonymous data, thereby this study is exempt from institutional review by the local Helsinki Committee.

## 3. Results

**Figure 1:**
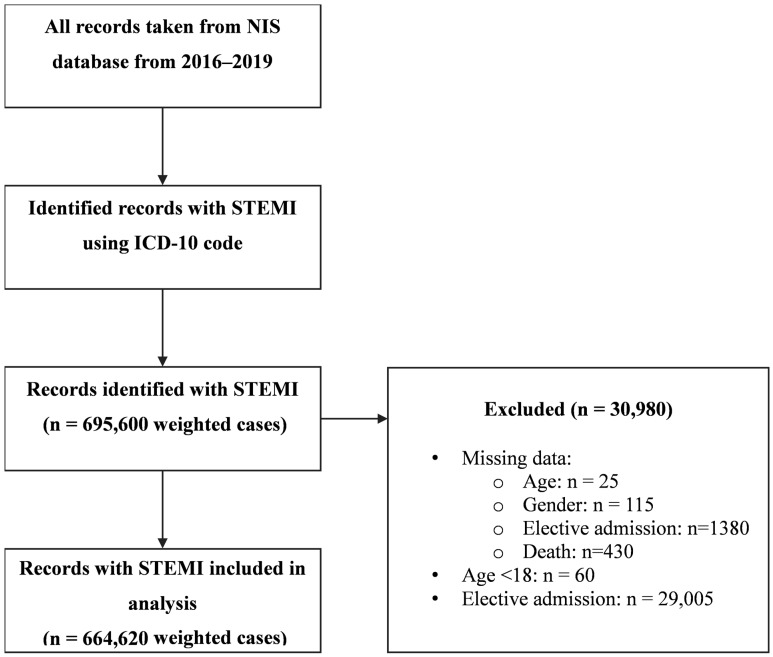
Flow diagram of the study population. STEMI = ST Elevation Myocardial Infraction; ICD International classification of diseases; NIS = national inpatient sample.

Between 2016 and the end of 2019, 695,600 individuals were hospitalized with the primary diagnosis of STEMI. Applying specific criteria for exclusion (Figure 1), a study cohort consisting of 664,620 patients was generated (0.04% excluded). Among these, 4905 (0.7%) were diagnosed with ICM sensitivity.

Differences in clinical characteristics on admission between the two groups are presented in Table 1. Patients with ICM sensitivity were older (median age 67 vs. 64, *p* < 0.001), more likely to be female (46.9% vs. 30.4%, *p* < 0.001), white (80.4% vs. 75.3%, *p* < 0.001), and have Medicare insurance (59.7% vs. 44.9%, *p* < 0.001). Patients with ICM sensitivity had a higher prevalence of comorbidities, including heart failure (26.6% vs. 24.7%, *p* < 0.001), valvular disease (10.8% vs. 8.4%, *p* < 0.001), atrial fibrillation/flutter (17.3% vs. 14.6%, *p* < 0.001), chronic renal failure (20.3% vs. 12.9%, *p* < 0.001), solid malignancies (2.4% vs. 1.8%, *p* = 0.001) and metastatic cancer (1.7% vs. 0.7%, *p* < 0.001), and were also more likely to have cardiovascular risk factors including diabetes (36.3% vs. 32.1%, *p* < 0.001), dyslipidemia (71.2% vs. 64.0%, *p* < 0.001), and smoking (55.9% vs. 51.9%, *p* < 0.001).

Differences in the management strategy and outcomes between the two groups are presented in Table 2. Patients with ICM sensitivity were less likely to undergo coronary angiography (90.6% vs. 91.5%, *p* = 0.022), PCI (76.9% vs. 79.6%, *p* < 0.001), or CABG surgery (4.2% vs. 5.2%, *p* = 0.002).

The primary endpoint of the study—in-hospital mortality was similar in both groups (7.2% vs. 7.9% for ICM-sensitive and non-ICM-sensitive, respectively) (*p* = 0.086). Acute ischemic CVA (1.3% vs. 1.4%, *p* = 0.741) and MACCE (12.0% vs. 12.0%, *p* = 0.951) were also similar among the two groups. Major bleeding was statistically lower in the ICM-sensitive group (2.4% vs. 3.3%, *p* < 0.001).

**Figure 2:**
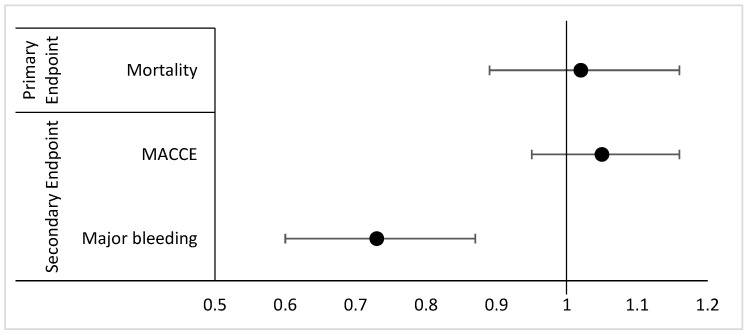
Forest plot depicting primary and secondary endpoints and 95% confidence intervals (CIs). The analysis is based on a multivariable logistic regression model, adjusted for baseline characteristics including age, gender, comorbidities, and cardiovascular risk factors. Each horizontal line represents the 95% confidence interval for the OR. The vertical line at OR = 1 indicates no difference between the groups. Values to the right of the line favor non-ICM-sensitive patients, while values to the left favor ICM-sensitive patients. Primary Endpoint: In-Hospital Mortality: OR 1.02 (95% CI: 0.89–1.16). Secondary Endpoint: MACCE: OR 1.05 (95% CI: 0.95–1.16), Major Bleeding: OR 0.73 (95% CI: 0.60–0.87).

Binary logistic regression for the outcomes between ICM-sensitive patients vs. non-ICM-sensitive patients was performed and is shown in Table 3 and displayed as a forest plot in Figure 2. The ICM-sensitive population had similar odds of in-hospital mortality (OR: 1.02, 95% CI: 0.89–1.16) and MACCE (OR: 1.05, 95% CI: 0.95–1.16). On the other hand, the ICM-sensitive population had lower odds of sustaining a major bleed (OR: 0.73, 95% CI: 0.60–0.87).

## 4. Discussion

Our study reports the prognosis of STEMI patients with iodinated contrast media sensitivity. To our knowledge, this is the largest study conducted thus far on this unique patient population. Treatment of these patients is urgent and premedication to prevent allergic reactions can be given only shortly prior to the procedure. Our results show that STEMI patients with and without ICM sensitivity had similar prognoses with regard to in-hospital mortality and MACCE. The ICM-sensitive population had on average a shorter length of stay, 3.87 days compared to 4 in the non-ICM-sensitive population, and lower rates of major bleeding.

Our series is the largest series published on this topic to date, encompassing 664,620 STEMI patients, of whom 4905 (0.7%) had a diagnosis of ICM sensitivity. Our incidence is similar to reports in previous studies. In most published large series, the reported prevalence of ICM sensitivity is not higher than 0.8% of the general population [15,16,17,18]. This percentage represents any patient who experienced some degree of symptoms.

Premedication to mitigate possible HSR for known susceptible patients with ICM sensitivity includes antihistamines and corticosteroids can be taken at 13, 7, 1 h before PCI [19,20]. A 2022 meta-analysis found that among 736 patients who had previously experienced ICM-induced adverse events, starting the premedication protocol helped reduce the incidence of adverse events by an OR 0.09 (0.03–0.25, CI 95%) [4]. This means that premedication administration before ICM administration is highly recommended in high-risk patients. In addition to premedication, switching the type of contrast media may also be effective in decreasing adverse event rates in known ICM-sensitive individuals, according to a 2018 Korean study [21].

In cases where it is not possible to wait, it has not been shown that giving any antihistamines or corticosteroids helps prevent recurrent events. In other words, when immediate treatment is needed, antihistamines or corticosteroids have not been shown to prevent recurrent events [22]. In our context, patients with STEMI cannot wait for premedication and need to be treated immediately. In the current study, we do not have information on what premedication, if any, was given and when was this administered, but our study found no signs of hazard in this population.

To further understand why individuals with ICM sensitivity have lower occurrences of significant bleeding, we can compare our findings to those from previous research on bleeding risks in patients receiving acute coronary syndrome (ACS) therapy. Fitchett et al. found that significant bleeding in ACS patients is associated with higher death and reinfarction rates. Reducing bleeding episodes is, therefore, crucial for improving patient outcomes [23].

In our study, the conservative management of ICM-sensitive patients who may undergo fewer invasive procedures like PCI or CABG could explain the reduced bleeding rates. This aligns with the principle of minimizing invasive procedures and antithrombotic exposure to lower bleeding risks.

Patients with STEMI who are at risk of bleeding are a distinct and challenging population to treat. Additional findings are required to make decisions about the risk–benefit ratio in patients with a high risk of bleeding who develop an inpatient STEMI [24]. When choosing medications among the currently available ones, an individualized therapy considering the risks and benefits may be more suitable [25]. These findings collectively emphasize the importance of individualized treatment strategies to minimize bleeding risks while ensuring effective management of STEMI patients, including those with ICM sensitivity.

We suggest that this may be attributed to the fact that more patients in the ICM-sensitive group were treated conservatively, maybe due to the fear of an HSR to ICM. Consequently, less bleeding occurred during and around the procedures, possibly due to less heparin anticoagulant treatment during PCI and administration of less potent anti-aggregate treatment in patients who did not undergo PCI.

Among our findings, it can be observed that ICM-sensitive patients tend to be older, are more likely to be of a white ethnicity, and are more likely to be female, and present with more cardiovascular risk factors (smoking, diabetes, and dyslipidemia) and have a higher number of comorbidities (heart failure, valvular disease, atrial fibrillation/flutter, chronic renal failure, solid malignancies, and metastatic cancer). Similar studies in the past have also found that ICM sensitivity is more common among women [16]. We did not find a study regarding differences between age and race groups. Recent papers about general allergic sensitization with respect to race have found, opposite to our finding, that black individuals tend to be sensitized more frequently than white individuals [26].

Both study groups had angiography in almost 91% of cases and PCI in 76–79%. Although ICM-sensitive patients statistically had fewer in-hospital procedures overall, the absolute rate is very high: 76.9% for ICM-sensitive vs. 79.6% for non-ICM-sensitive. This small diffidence is likely due to medical teams considering the risk–benefit ratio for their patients, and not wanting to expose their patients to a substance that could cause HSR. Unfortunately, the NIS database does not contain specific information about any premedication regimen, such as corticosteroids and antihistamines, given to ICM-sensitive patients before a procedure.

Another point to take into consideration is that an individual may think they are ICM-sensitive due to experiencing some past allergy-like symptoms after a previous test, even though they are not truly ICM-sensitive. This may mask the prognosis of true ICM-sensitive patients. Similarly, a study regarding perceived penicillin allergy showed that around 90% of these people are not genuinely allergic and may safely use ß-lactam antibiotics [27].

Severe ICM allergy may induce respiratory arrest, cardiac arrest, pulmonary edema, convulsions, and cardiogenic shock, which are life-threatening and can end in lasting morbidity or mortality if not handled properly and treated with IV Epinephrine [2]. Fortunately, severe reactions are rare, occurring in between 0.04% and 0.004% of patients [3].

### Limitations

While this study provides valuable insights regarding the relationship between ICM sensitivity and the prognosis of patients with STEMI, it is important to acknowledge several limitations. It is still important to note that although we did not manage to prove that ICM-sensitive patients are at risk for a worse prognosis, our findings are relevant only to our population.

The ICD-10 code used to filter ICM-sensitive patients does not detail the severity of allergy-like symptoms. If this was available, it could have provided additional insight into the relationship.

Importantly, the NIS database does not provide information about when the ICM-sensitive diagnosis was made. Did the patient have the diagnosis prior to the hospitalization or was the allergy diagnosed following an HSR during the current hospitalization? In addition, the patient’s pre-medication regimen as well as any complementary treatments such as steroids, aspirin or P2Y12 inhibitors given during hospitalization, were not documented in this study.

Our study is a retrospective observational study and, as such, there is room for further studies to confirm our observation and examine possible reasons to have lower odds of major bleeding and a shorter average length of stay among ICM-sensitive patients.

## 5. Conclusions

In this large cohort, 0.7% of patients had an ICM-sensitive diagnosis, consistent with previous studies. These patients were typically older, white females with more comorbidities and cardiovascular risk factors. Both ICM-sensitive and non-ICM-sensitive groups underwent angiography in 90% of cases, with PCI performed in 76–79% of all patients. ICM-sensitive patients were managed more conservatively and were slightly less likely to undergo PCI or CABG. Despite similar odds of mortality or MACCE, ICM-sensitive patients had lower odds of major bleeding and a shorter average length of stay. However, severe allergic reactions can still be fatal; therefore, careful clinical judgment is required for STEMI patients with known ICM sensitivity undergoing urgent PCI.

## Figures and Tables

**Table 1:** Demographics, record characteristics and comorbidities of patients, stratified by ICM sensitivity status.

	Non-ICM-Sensitive	ICM-Sensitive	*p*-Value
Number of weighted records	659,715 (99.3%)	4905 (0.7%)	
Age (Mean, Median, IQR)	64 (63, 18)	67 (67, 17)	
Gender			<0.001
Male	69.6%	53.1%	
Female	30.4%	46.9%	
Race (uniform)			<0.001
White	75.3%	80.4%	
Black	8.9%	10.2%	
Hispanic	8.7%	5.4%	
Asian or Pacific Islander	3.0%	1.6%	
Native American	0.5%	0.5%	
Other	3.6%	1.8%	
Region of hospital			0.03
Northeast	17.2%	18.6%	
Midwest	22.8%	22.8%	
South	39.8%	40.3%	
West	20.2%	18.3%	
Location/teaching status of hospital (STRATA)			0.153
Rural	6.1%	6.0%	
Urban nonteaching	22.6%	21.5%	
Urban teaching	71.3%	72.5%	
Median household income national quartile for patient ZIP Code			<0.001
1st	28.1%	32.0%	
2nd	27.2%	27.4%	
3rd	24.6%	23.1%	
4th	20.1%	17.5%	
Primary expected payer (uniform)			<0.001
Medicare	44.9%	59.7%	
Medicaid	10.6%	9.4%	
Private insurance	33.9%	23.6%	
Self-pay	6.8%	3.4%	
No charge	0.6%	0.3%	
Other	3.2%	3.7%	
Record Characteristics			
Cardiac Arrest	6.2%	4.2%	<0.001
Ventricular Fibrillation	8.4%	4.8%	<0.001
Ventricular Tachycardia	12.3%	11.0%	0.006
Cardiogenic Shock	13.7%	10.2%	<0.001
Length of stay, (days), median (IQR)	4 (2, 2)	3.87 (3, 2)	
Total charge, (USD), median (IQR)	116,369 (82,872, 76,212)	111,378 (83,429, 76,776)	
Comorbidities			
Previous MI	11.8%	17.8%	<0.001
Cerebrovascular disease	2.6%	4.3%	<0.001
Heart failure	24.7%	26.6%	<0.001
Valvular disease	8.4%	10.8%	<0.001
Atrial fibrillation/flutter	14.6%	17.3%	<0.001
Hypertension	72.6%	82.3%	<0.001
Dyslipidemia	64.0%	71.2%	<0.001
Diabetes Mellitus	32.1%	36.3%	<0.001
Smoker	51.9%	55.9%	<0.001
Peripheral vascular disease	6.1%	10.5%	<0.001
Chronic lung disease	12.2%	16.5%	<0.001
Chronic renal failure	12.9%	20.3%	<0.001
Obesity	17.2%	18.8%	0.004
Anemia	14.8%	17.4%	<0.001
Thrombocytopenia	4.1%	4.0%	0.705
Coagulopathy	1.9%	2.3%	0.012
Dementia	3.3%	4.0%	0.011
Chronic Liver Disease	0.3%	0.4%	0.408
Solid malignancy	1.8%	2.4%	0.001
Hematologic Malignancies	0.7%	1.1%	<0.001
Metastatic cancer	0.7%	1.7%	<0.001

**Table 2:** In-hospital procedures and outcomes, stratified by ICM-sensitive status.

	Non-ICM-Sensitive	ICM-Sensitive	*p*-Value
Number of weighted records	659,715 (99.3%)	4905 (0.7%)	
In-hospital procedures			
Coronary Angiography	91.5%	90.6%	0.022
PCI	79.6%	76.9%	<0.001
CABG	5.2%	4.2%	0.002
Thrombolysis	0.6%	0.3%	0.015
Circulatory support (inc. IABP, LV assist device and ECMO)	10.5%	9.1%	0.001
Mechanical Ventilation	11.2%	7.8%	<0.001
In-hospital Outcomes			
MACCE	12.0%	12.0%	0.951
Mortality	7.9%	7.2%	0.086
Acute Ischemic CVA	1.4%	1.3%	0.742
Major Bleeding	3.3%	2.4%	0.001
○ GI bleed	1.9%	1.4%	0.025
○ Procedural related bleeding	1.1%	0.7%	0.018
○ Retroperitoneal Bleed	0.2%	0.3%	0.065
○ Intracranial Hemorrhage	0.3%	0.2%	0.199

**Table 3:** Binary logistic regression for outcomes for ICM-sensitive patients vs. non-ICM-sensitive patients. Variables entered on step 1: Age in years at admission, Indicator of sex, Race, Region of hospital, Location/teaching status of hospital, Median household income national quartile for patient ZIP Code, Primary expected payer, Cardiac Arrest, Ventricular Fibrillation, Ventricular Tachycardia, Cardiogenic shock, Length of stay, Total charges, Previous MI, Cerebrovascular disease, Heart failure, Valvular disease, Atrial fibrillation/flutter, Hypertension, Dyslipidemia, Diabetes Mellitus, Smoker, Peripheral vascular disease, Chronic lung disease, Chronic renal failure, Obesity, Anemia, Thrombocytopenia, Coagulopathy, Dementia, Chronic Liver Disease, Solid malignancy, Hematologic Malignancies, Metastatic cancer, Contrast allergy.

	OR (95% CI)	OR *p*-Value
Mortality	1.02 (0.89–1.16)	0.798
MACCE	1.05 (0.95–1.16)	0.355
Major bleeding	0.73 (0.60–0.87)	0.001

## Data Availability

The data that support the findings of this study are available from HCUP (https://hcup-us.ahrq.gov). Restrictions apply to the availability of these data, which were used under license for this study.

## Supplementary Materials

The following supporting information can be downloaded at: https://www.mdpi.com/article/10.3390/medicina60060973/s1, Table S1. ICD 10 codes used to extract data.
