# Supplementary material for: The Effect of Iodinated Contrast Media Sensitivity on the Prognosis of Patients with STEMI
Source: Medicina (Kaunas). 2024 Jun 12;60(6):973. doi: 10.3390/medicina60060973 (PMC11205422; doi:10.3390/medicina60060973)
Supplement: Supplementary file 1 [file medicina-60-00973-s001.zip › medicina-3004681-supplementary.pdf]

Table S1. ICD 10 codes used to extract data.

Variable	Codes
**Patient and Record Characteristics**	
Ischemic Heart disease	I25.2; I25.5; Z95.1; I25.7x.; Z98.61; Z95.5
Cerebrovascular disease	I65-69
Chronic renal failure	N18
Aortic Disease	I70.0
Peripheral Vascular Disease of extremities	I70.2x-I70.9x; I73.x; Z98.62
STEMI	I21.0-3
Cardiac Arrest	I46.x
Ventricular Fibrillation	I49.01
Ventricular tachycardia	I47.2
Cardiogenic Shock	R57.0
HFrEF	I50.2
HFpEF	I50.3
Valvular disease	I05-08; I34-37
Atrial fibrillation/flutter	I48
Hypertension	I10-16
Dyslipidaemia	E78
Diabetes	E08-13
Smoking	Z87.891 , Z72.0
Chronic lung disease	J40-47
Obesity	E66
Anaemia	D55-59
Thrombocytopenia	D69.3-.6
Coagulopathy	D65-68;D69.0-.2
Dementia	F01-03
Chronic Liver Disease	K73-74
Homelessness	Z59.0
Solid malignancy	C00.x-C76.x; C80.x
Hematologic Malignancies	C81-96
Metastatic cancer	C77.x-79.x
Contrast agent allergy	Z91.041
**In Hospital Procedures**	
Coronary Angiography	B211x
PCI	02703x/13x/23x/33x
CABG	02100*/04*/10*/14*/20*/24*/30*/34*
Thrombolysis	3E07317

Circulatory support (inc. IABP, LV assist device and ECMO).	5A02x, 5A1522G, 5A15A2G, 5A15A2H
Mechanical Ventilation	5A19054/35Z/45Z/55Z
**In Hospital Outcomes**	
Acute Ischemic CVA	I63
Coronary artery dissection	I2542
Pericardial effusion (incl. tamponade)	I23.0 I31.2 I31.4 I31.3
Tamponade	I31.4
Dressler's syndrome	I24.1
Post MI angina	I23.7
Intracardiac Thrombus	I23.6
Mechanical complications	I23.1-I23.5
GI bleed	K92.0-92.2; K25.0-25.2; K25.4-25.6; K26.0-26.2; K27.0-27.2; K27.4-27.6; K28.0-28.2; K28.4-28.6
Retroperitoneal Bleed	K66.1
Intracranial Hemorrhage	I60-62
